# Expression of IL-27 by Tumor Cells in InvasCutaneous and Metastatic Melanomas

**DOI:** 10.1371/journal.pone.0075694

**Published:** 2013-10-10

**Authors:** Julie Gonin, Agnès Carlotti, Céline Dietrich, Anne Audebourg, Brigitte Radenen-Bussière, Anne Caignard, Marie-Françoise Avril, Marie-Cécile Vacher-Lavenu, Frédérique Larousserie, Odile Devergne

**Affiliations:** 1 Centre National de la Recherche Scientifique Unité Mixte de Recherche 8147, Université Paris Descartes, Sorbonne Paris Cité, Paris, France; 2 Service d’Anatomie Pathologique and Université Paris Descartes, Assistance Publique des Hôpitaux de Paris, Hôpital Cochin, Paris, France; 3 Institut Cochin, Institut National de la Santé et de la Recherche Médicale U1016, Centre National de la Recherche Scientifique Unité Mixte de Recherche 8104, Université Paris Descartes, Paris, France; 4 Service de Dermatologie, Assistance Publique des Hôpitaux de Paris, Hôpital Cochin, Paris, France; University of Tennessee, United States of America

## Abstract

Interleukin (IL)-27 is a cytokine of the IL-12 family that displays either immunostimulatory or immunosuppressive functions depending on the context. In various murine tumor models including melanoma models, ectopic expression of IL-27 has been shown to play an anti-tumoral role and to favor tumor regression. In this study, we investigated whether IL-27 might play a role in the development of melanoma in humans. We analyzed the *in situ* expression of IL-27 in melanocytic lesions (n = 82) representative of different stages of tumor progression. IL-27 expression was not observed in nevus (n = 8) nor in *in situ* melanoma (n = 9), but was detected in 28/46 (61%) cases of invasive cutaneous melanoma, notably in advanced stages (19/23 cases of stages 3 and 4). In most cases, the main source of IL-27 was tumor cells. Of note, when IL-27 was detected in primary cutaneous melanomas, its expression was maintained in metastatic lesions. These *in situ* data suggested that the immunosuppressive functions of IL-27 may dominate in human melanoma. Consistent with this hypothesis, we found that IL-27 could induce suppressive molecules such as PD-L1, and to a lesser extent IL-10, in melanoma cells, and that the *in situ* expression of IL-27 in melanoma correlated with those of PD-L1 and IL-10.

## Introduction

Malignant melanoma, the incidence of which has been constantly rising over the past decades, is among the most aggressive human tumors. Whereas primary tumor at early stages is curable by complete surgical excision, metastatic melanoma is often resistant to standard therapies such as conventional chemotherapy regimens [1].

Because malignant melanoma is a highly immunogenic tumor, immunotherapeutic approaches, such as cytokine administration to stimulate the anti-tumoral response and restrict tumor progression, have drawn interest. Recently, a member of the IL-12 family, IL-27, has been proposed as a candidate for anti-tumoral therapy, notably in melanoma [2], [3]. IL-27 is a heterodimeric cytokine composed of two subunits, EBI3 and p28 [4], [5]. It is expressed at high levels by activated macrophages and dendritic cells and displays broad immunological functions (reviewed in ref. [6]). *In vitro* studies and mouse models have suggested that it may play a potent anti-tumoral role (reviewed in ref. [7]). First, recombinant mouse or human IL-27 has been shown to promote the *in vitro* generation of CD8+ cytotoxic T cells (CTL) [8], [9]. Second, in mice, administration of an IL-27 expression plasmid resulted in an adjuvant activity for *in vivo* generation of Ag-specific CTL [10] and in improved tumor eradication [11]. In addition, in various tumor models, including C26 colon carcinoma cells [12], [13], Lewis lung carcinoma [14], TBJ neuroblastoma [15], [16], and B16F10 melanoma cells [17], [18], tumor cell lines genetically engineered to overexpress IL-27 showed growth inhibition, *in vivo*, compared to the IL-27-negative parental cell lines. This inhibiting effect was ascribed to an effect of IL-27 on various immune cells including CD8+ T cells, CD4+ T cells or NK cells, an anti-angiogenic effect, or direct suppressive effects on tumor cells [12]–[19].

Whether endogenous IL-27 plays a physiological role in the development of human melanoma is unknown. Melanoma originates from melanocytes. These cells are mainly present in the skin, where they are located in the basal layer of the epidermis. Tumor progression from normal melanocyte to malignant melanoma is a multistep process characterized by distinct histologic features. In this study, we investigated the *in situ* expression of IL-27 in melanocytic lesions representative of different stages of tumor progression. Unexpectedly, we observed that IL-27 expression in melanomas was not associated with tumor regression, but instead with tumor progression. This finding led us to investigate the effect of IL-27 on the induction of immunosuppressive molecules by melanoma cells in *in vitro* experiments.

## Materials and Methods

### Ethics statement

Studies on human tissues were conducted in accordance with the declaration of Helsinki and were approved by the institutional review board of Cochin Hospital. These studies were performed retrospectively on fixed biopsies that had been collected for diagnosis purpose. The need for written consent of the patient for subsequent immunohistochemical studies was waived by the hospital institutional review board. Samples were analyzed anonymously. Studies on human melanoma cells were approved by « Ile de France » ethics committee, and the declaration of Helsinki protocols were followed.

### Tissue biopsies

All tissues analyzed in this study were retrieved from the files of the Department of Pathology of Cochin Hospital (Paris). Skin biopsies included cases of benign nevus (n = 8, all compound nevi), melanoma *in situ* (n =  9), and primary invasive cutaneous melanoma (n = 46). Cases of primary invasive cutaneous melanoma were classified based on tumor thickness (Breslow index) according to the current American Joint Committee on Cancer (AJCC) staging system [20]. Eight cases were of stage 1 (thickness <1 mm), 12 of stage 2 (1.01–2.0 mm), 12 of stage 3 (2.01–4.0 mm), 11 of stage 4 (>4 mm), and 3 could not be staged. They included superficial spreading melanoma (n = 30), nodular melanoma (n = 8), acral lengitinous melanoma (n = 3), lentigo maligna melanoma (n = 1) and unclassified cases (n = 4). Nineteen cases of metastatic melanoma, 15 of which came from the same patients as the ones with primary cutaneous melanoma, were also included. Metastatic melanoma involved lymph nodes in 18 cases and adrenals in one case. None of the patients had received therapy at the time of the biopsy.

### Immunohistochemistry

Immunostaining was performed on serial tissue sections from formalin-fixed paraffin-embedded tissues. EBI3 was detected using 2G4H6 mouse mAb (IgG2a) [21], in parallel with an isotype-matched control mAb (UPC10, IgG2a, ICN Pharmaceuticals). p28 was detected using affinity-purified rabbit polyclonal Abs directed against a N-terminal peptide of p28 (gift from Stefan Pflanz and Robert Kastelein, Merck Biosciences, Palo Alto), in parallel with normal rabbit IgG (Sigma) as a negative control. The characteristics of the anti-EBI3 and anti-p28 Abs and their use for immunohistochemical studies were previously described [21]–[23]. Expression of IL-27 requires co-expression of both EBI3 and p28 within cells. Therefore, in most cases, tissues were first tested for EBI3 and, when positive, also tested for p28. Tumor cells were scored as positive for IL-27 only when both subunits were co-expressed. PD-L1 was detected using affinity-purified rabbit polyclonal Abs (#4059, ProSci Inc.) and IL-10 using affinity-purified goat polyclonal Abs (R&D Systems). Melan-A Ab (clone A103) was purchased from Dako.

Sections were dewaxed in xylene, rehydrated and subjected to Ag retrieval by heat pretreatment using citrate buffer. For immunostaining with rabbit or goat Abs, slides were first saturated with TBS containing 20% normal human serum prior to incubation with the primary Abs. After a 30 min incubation with the primary Abs, slides were incubated with anti-mouse or anti-rabbit SuperSensitive link (BioGenex), or biotinylated anti-goat Abs (Vector laboratories), followed by incubation with alkaline phosphatase-labelled streptavidine (SuperSensitive label, BioGenex). Alkaline phosphatase activity was developed using permanent red (Sigma-Aldrich) as chromogen. Sections were counterstained with Mayer hematoxylin. Images were captured on a NanoZoomer 2.0-RS slide scanner (Hamamatsu Corporation) and processed with NDP Viewer.

### Cell culture

Melanoma cell lines used in this study included A375, SK-Mel-28 (purchased from ATCC), Mel-S and Mel-C (derived from metastatic lymph nodes in the laboratory of Dr Anne Caignard [24]). Cell lines were maintained in RPMI 1640 medium supplemented with 10% fetal bovine serum, 1% glutamine and 1% antibiotics. They were cultured for various times in the absence or presence of IL-27 (100 ng/ml, R&D Systems) and in some cases TGF-ß1 (0.1 ng/ml, R&D Systems), before analysis by quantitative real time PCR (qPCR) or FACS. Peripheral blood mononuclear cells (PBMC) were purified from adult healthy donors (Etablissement Français du Sang, Paris ; convention #07/CABANEL/106) by Ficoll-Paque Plus (Amersham Biosciences) gradient centrifugation. In some cases, A375 cells (3×10^4^ per well) were pre-incubated in 48-well plate in the absence or presence of IL-27 for 15 hrs, washed extensively, and incubated for 3 days with PBMC (3×10^5^ per well) in the presence of CD3/CD28 activation beads (1 bead per 2 cells, Miltenyi Biotech). A neutralizing anti-PD-L1 Ab (clone MIH1, e.Bioscience) or an isotype-matched control Ab (MOPC21) were added to the culture at 10 µg/ml. Cell culture supernatants were collected for ELISA.

### Gene expression analysis

DNA-free total RNA was isolated by using RNeasy Plus Micro kit (Qiagen). RNA was reverse transcribed into cDNA using M-MLV reverse transcriptase and random hexamer primers (Invitrogen). qPCR for PD-L1 (CD274) and IL-10 was performed using pre-designed TaqMan® gene expression assays and Taqman Universal PCR Master mix (Applied Biosytems). For each sample, triplicate reactions were run for 40 cycles on Step One Plus thermal cycler (Applied Biosystems). Levels of target mRNA were normalized relative to levels of ß2-microglobulin mRNA or 18S RNA, and relative gene expression was calculated using the comparative cycle threshold method.

### FACS analysis

For cell surface staining, cells were saturated in PBS containing 20% normal human serum before incubation with specific Abs. All staining and washes were performed in PBS containing 2% fetal bovine serum and 0.01% sodium azide. The following conjugated mouse mAbs were used: CD4-APC (BD Biosciences), CD8-APC (BD Biosciences), CD16-FITC (Immunotech), CD56-PC5 (Beckman-Coulter), anti-gp130-PE mAb (BD Biosciences), anti-IL-27Rα-PE mAb (R&D Systems), and anti-PD-L1-PE mAb (eBioscience), in parallel with the proper PE-conjugated isotype control mAbs. Cells were analyzed on FACSCanto II and data were analyzed using FlowJo software (TreeStar).

### ELISA

Supernatants from melanoma cell lines or from co-culture experiments were tested for IL-10 or IL-2 using Quantikine ELISA kit (R&D Systems).

### Statistical analysis

Unless otherwise indicated, a Fisher exact test was used for statistical analysis.

## Results

### Lack of IL-27 expression in benign melanocytic lesions

**Figure 1:**
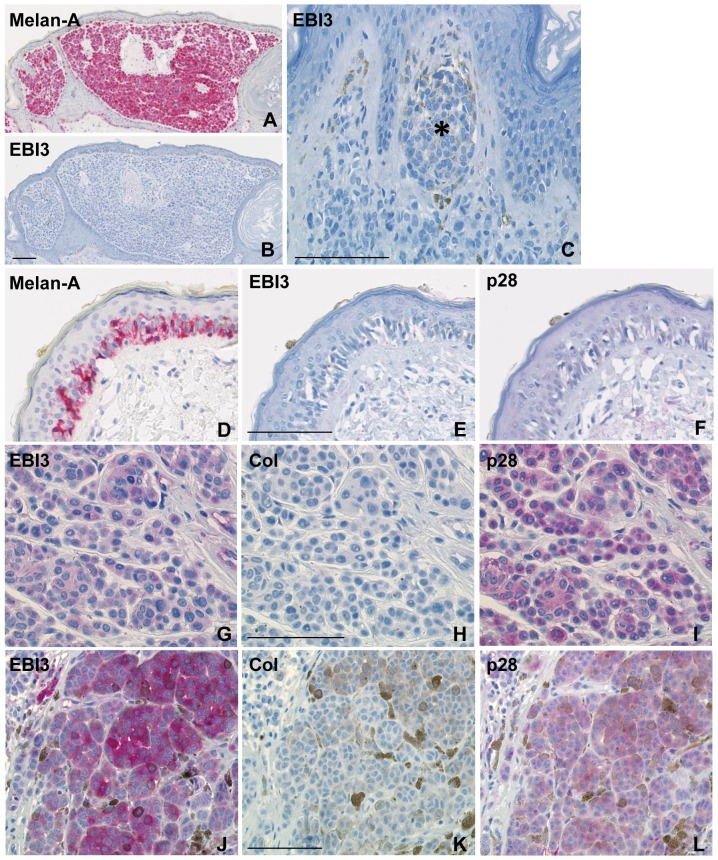
Immunohistochemical analysis of IL-27 expression in nevus, melanoma *in situ* and primary invasive cutaneous melanoma. (A-B) Serial sections of a case of nevus were analyzed for Melan A, a marker of melanocytic cells, or for EBI3. Melan-A-positive cells are all negative for EBI3. (C) A nest of melanocytic cells (asterisk) negative for EBI3, from another case of nevus, is shown at a higher magnification. (D-F) Serial sections of a case of melanoma *in situ* were analyzed for Melan A, EBI3 and p28. Melan-A positive tumor cells are negative for both EBI3 and p28. (G-L) : Serial sections of 2 cases (one case per line) of primary invasive cutaneous melanoma were stained with anti-EBI3, anti-p28 or control isotype Ab (the control for rabbit Abs is shown). Co-expression of EBI3 and p28 is observed in tumor cells. The bar represents 100 µM.

Melanocytic nevi are considered to represent the benign counterpart of melanoma. They are formed by melanocytes that proliferate and aggregate, leading to the formation of nests or clusters of cells located at the epidermis-dermis junction and/or in the dermis. EBI3 immunohistochemical analysis of skin biopsies from patients with nevi (n = 8) showed that, apart from rare EBI3-positive cells exhibiting morphological features of dendritic cells and located in the dermis, all cases were virtually negative for EBI3. In particular, nevus cells localized in junctional or intradermal nevi were negative for EBI3 (Figure 1A-C). This lack of EBI3 detection indicates that IL-27 is not expressed at detectable levels in nevi.

### Lack of IL-27 expression in non-invasive melanomas

The first and least aggressive stage of malignant melanoma is the melanoma *in situ*, that grows laterally and is confined to the epidermis. Of 9 cases of melanoma *in situ*, 8 cases were largely negative for EBI3 (Figure 1D-F). In only one case, malignant cells stained positive for EBI3. However, no staining for p28 was observed in this case, as in the 8 other cases, indicating that the malignant cells do not express IL-27. Thus, as observed in nevus, no substantial amounts of IL-27 were detected in melanoma *in situ*.

### Expression of IL-27 by tumor cells in primary invasive cutaneous melanomas

The subsequent step of melanoma progression is characterized by vertical growth of tumor cells and invasion of the dermis to a variable extent. At this stage, tumor cells acquire metastatic potential.

**Figure 2:**
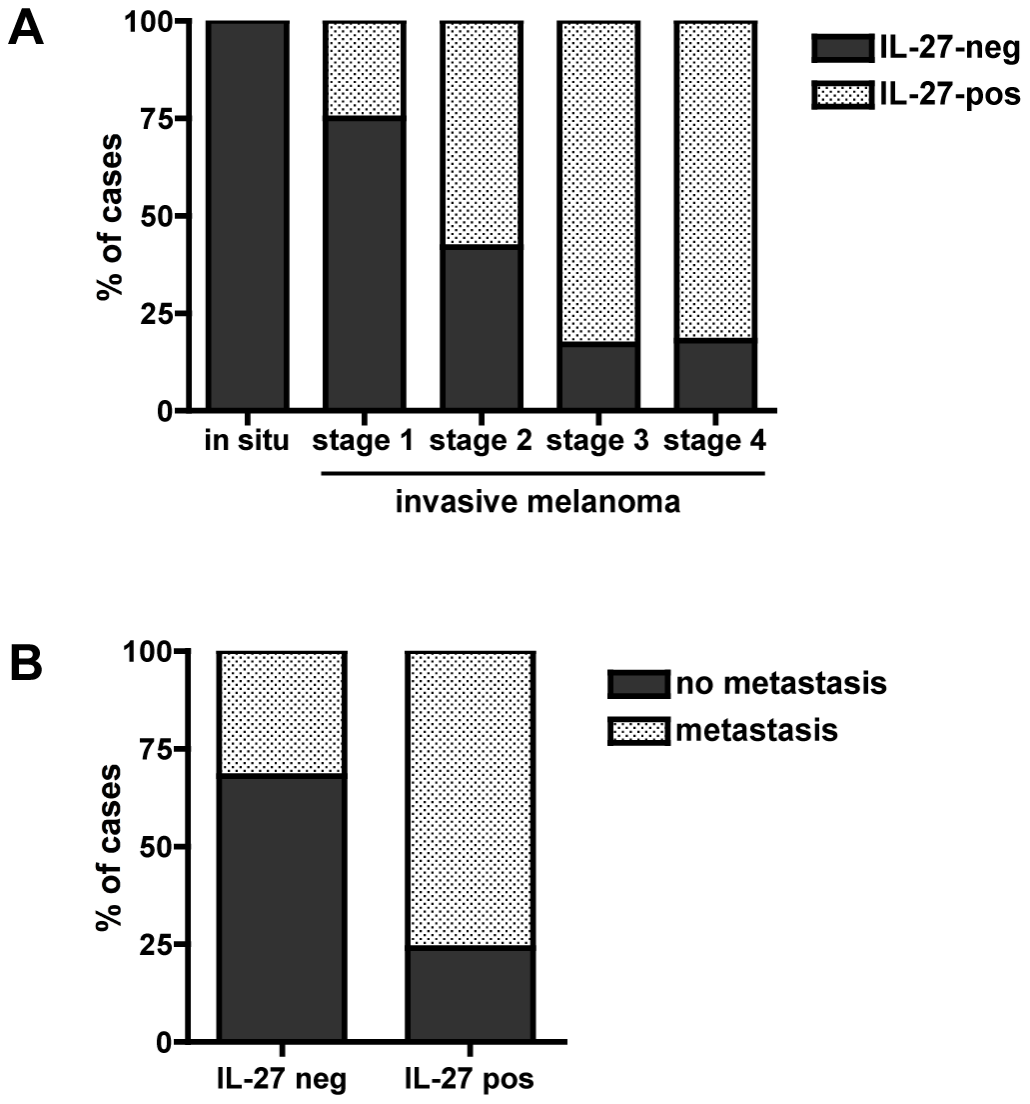
Correlation between IL-27 expression by tumor cells and the histological stage or the occurrence of metastasis. (A) Percentage of IL-27-negative or -positive cases depending on the histological stage of the melanoma. (B) Distribution of cases that metastasized or not among IL-27-negative or -positive cutaneous melanomas (*in situ* and invasive).

We analyzed IL-27 expression in 46 cases of invasive cutaneous melanomas. In these cases, a heterogeneous pattern of EBI3 and p28 staining was detected. Several types of cells stained positive for both EBI3 and p28. Variable numbers of macrophages/melanophages located in the dermis were positive for EBI3 and p28 (not shown). As previously observed in lymphoid tissues [22], [23], a weak EBI3 and p28 staining could be observed in endothelial cells (not shown). Interestingly, positivity for EBI3 and p28 was observed in tumor cells (Figure 1G-L). These latter accounted for the majority of positive cells in most cases. This tumor cell staining was heterogeneous among cases. It was observed independently of the morphology of tumor cells, large or small, round or spindle, and of the histological type. Positivity for IL-27 in tumor cells was observed in 2/8 cases of stage 1, 7/12 cases of stage 2, 10/12 cases of stage 3, and 9/11 cases of stage 4 (Figure 2A). Thus, although IL-27 positivity was not restricted to a specific stage of melanoma, it was found in a higher proportion of advanced primary melanomas (19/23 cases of stages 3 and 4) compared to early lesions (9/20 cases of stages 1 or 2).

A 4-year clinical follow-up was available for 43 of the patients with primary cutaneous melanomas (*in situ* or invasive) and indicated that 23 developed metastases, whereas 20 did not. Expression of IL-27 by tumor cells was observed in 16/23 cases (70%) that developed metastases, while it was not found in 15/20 cases (75%) that did not (p<0.01) (Figure 2B).

### IL-27 expression by tumor cells is maintained in metastases

**Figure 3:**
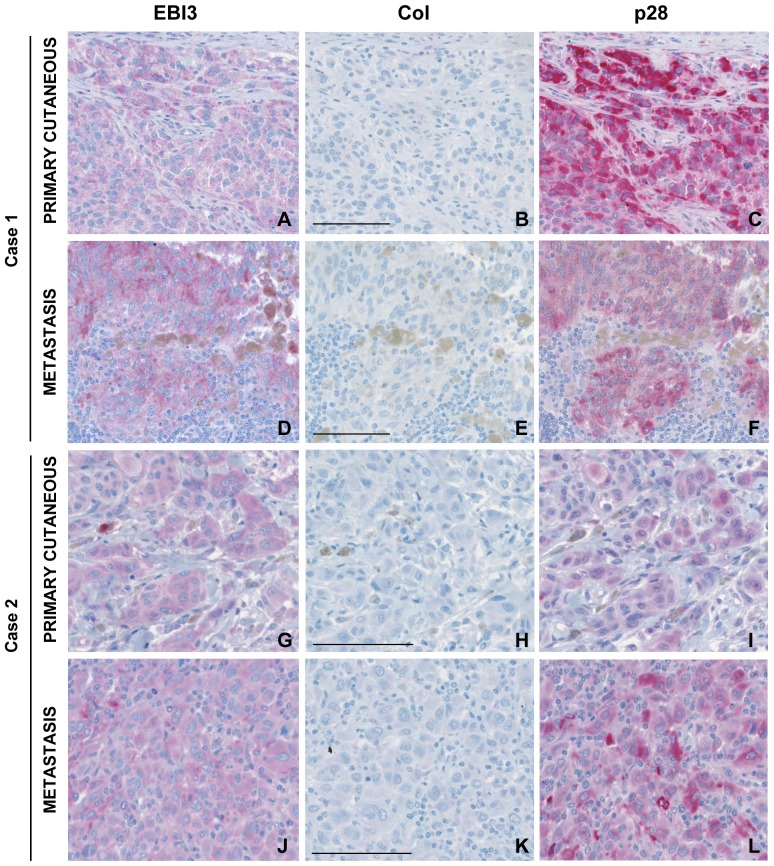
Immunohistochemical analysis of IL-27 expression in primary and metastatic melanoma from two patients. Serial sections from the primary cutaneous melanoma and the corresponding metastasis from two patients were stained with anti-EBI3, anti-p28 or control Abs. The expression of IL-27 subunits observed in tumor cells in the primary melanoma is maintained in the metastasis. The bar represents 100 µM.

We also analyzed cases of metastatic melanoma (n = 19) for IL-27 expression. These cases included 15 patients for which the primary cutaneous melanoma was previously analyzed for IL-27 expression. Expression of EBI3 and p28 by tumor cells was detected in most cases (17/19 cases, 89%). Interestingly, comparison of IL-27 staining in primary and metastatic melanoma from the same patient showed that expression of EBI3 and p28 by tumor cells was not downregulated upon tumor progression (Table I and Figure 3). Indeed, when positivity for IL-27 was observed in tumor cells in the primary cutaneous tumor (10/15 cases), it was maintained in the metastatic melanoma (Figure 3). In addition, in 4 of the 5 patients for which the primary cutaneous melanoma was negative for IL-27 expression, an upregulation of IL-27 expression was observed in the metastatic lesion (Table I). Taken together, these data further indicate that IL-27 expression by tumor cells is associated with tumor progression.

**Table I:** Analysis of IL-27 expression in primary cutaneous and metastatic melanoma from single patients.

	IL-27+ tumor cells	
	PRIMARY TUMOR	METASTASIS
**Stage 1**	–	+
**Stage 2**	+	+
	+	+
	–	+
**Stage 3**	+	+
	+	+
	+	+
**Stage 4**	+	+
	+	+
	+	+
	–	+
	+	+
	–	–
**Unclassified**	–	+
	+	+

### Analysis of IL-10 and PD-L1 induction by IL-27 in melanoma cells

Because IL-27 expression in melanoma appeared to correlate with tumor progression, we investigated by which mechanisms IL-27 could favor melanoma progression.

**Figure 4:**
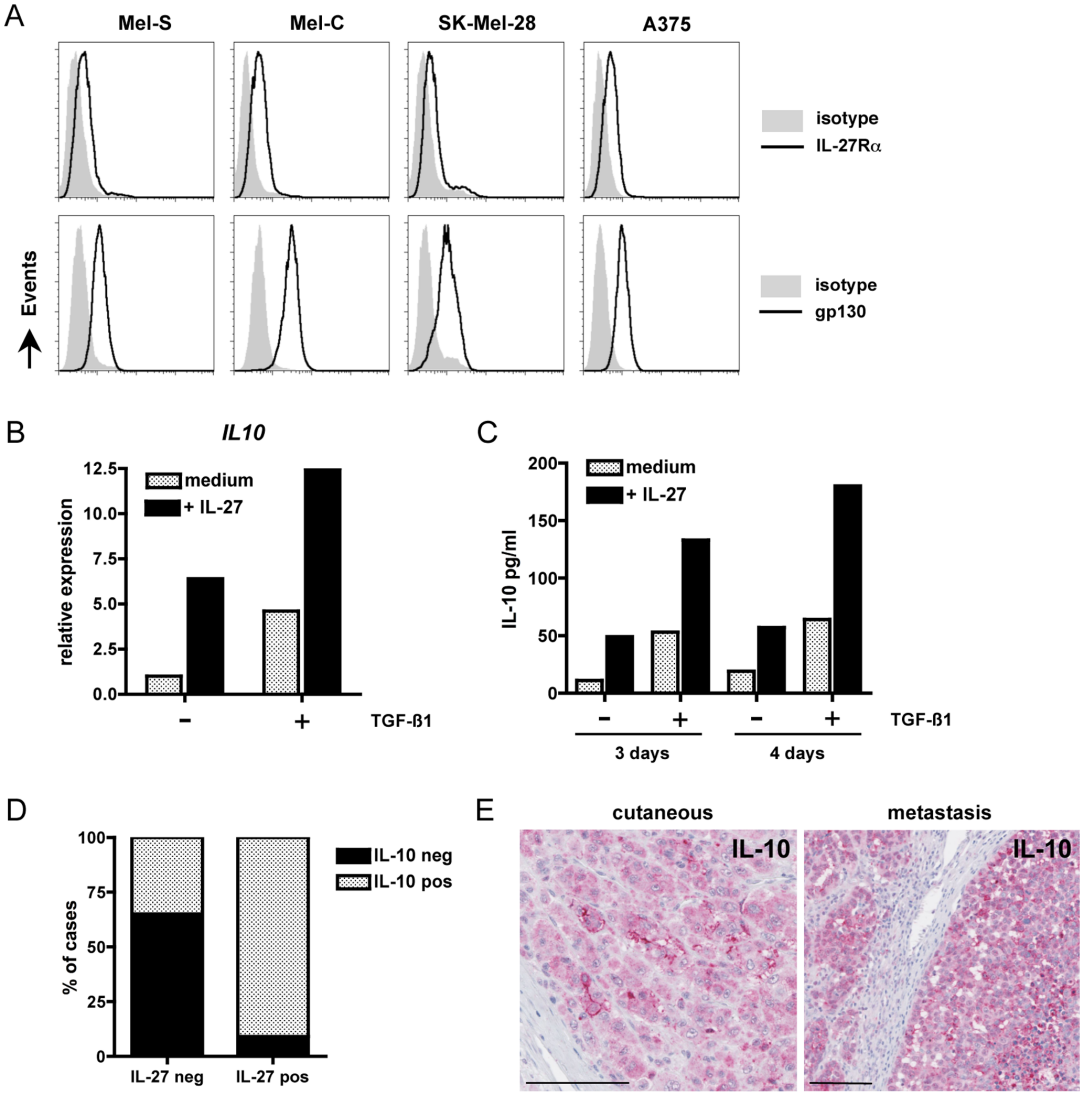
Analysis of IL-10 induction by IL-27 in melanoma. (A) Constitutive expression of IL-27Rα and gp130 was analyzed by FACS on 4 melanoma cell lines as indicated. (B) Expression of *IL-10* was analyzed by qPCR in the A375 melanoma cell line cultured for 3 days in the presence or absence of IL-27 or TGF-ß1. (C) Supernatants from the A375 cell line cultured for 3 and 4 days in the presence or absence of IL-27 or TGF-ß1 were tested for IL-10 by ELISA. A representative experiment of 3 is shown in (B) and (C). (D) Distribution of cases that expressed IL-10 or not, among IL-27-negative or -positive cutaneous and metastatic melanomas. (E) Staining for IL-10 in a case of a primary cutaneous melanoma and a case of metastatic melanoma that were positive for IL-27. The bar represents 100 µM.

Tumor cells, including melanoma cells, are known to express various suppressive molecules to escape the immune response. A major suppressive function of IL-27 has been ascribed to its ability to induce IL-10 production in various T cell subsets, including murine and human CD4+ and CD8+ T cells [25]–[28]. In murine CD4+ T cells, this effect is potentiated in the presence of TGF-ß [27]. Both IL-10 and TGF-ß have been previously shown to be expressed in melanoma [29]. In particular, IL-10 expression by tumor cells correlates with tumor progression [30], [31]. To test the hypothesis that IL-27 could promote melanoma progression by inducing IL-10 expression in melanoma cells, we tested the effect of exogenous recombinant IL-27, alone or in combination with TGF-ß1, on IL-10 expression in 4 melanoma cell lines that express the two subunits of the IL-27 receptor, IL-27Rα and gp130 (Figure 4A), but do not express IL-27 (not shown). In 1 of the 4 cell lines, the A375 cell line, we observed that IL-27 induced IL-10, both at the mRNA and protein level, and that this effect was enhanced in the presence of TGF-ß1 (Figure 4B and C). Thus, it is conceivable that IL-27 produced by tumor cells could contribute to IL-10 induction in an autocrine manner, in some cases. To evidence a possible correlation between IL-10 and IL-27 expression in melanoma lesions, we analyzed by immunohistochemistry IL-10 expression in 43 cases of primary cutaneous or metastatic melanoma. Consistent with the *in vitro* findings, a higher frequency of IL-10-positive cases was observed among IL-27-positive cases (21/23 cases, 91%) than among IL-27-negative cases (7/20, 35%) (p<0.001) (Figure 4D and E).

**Figure 5:**
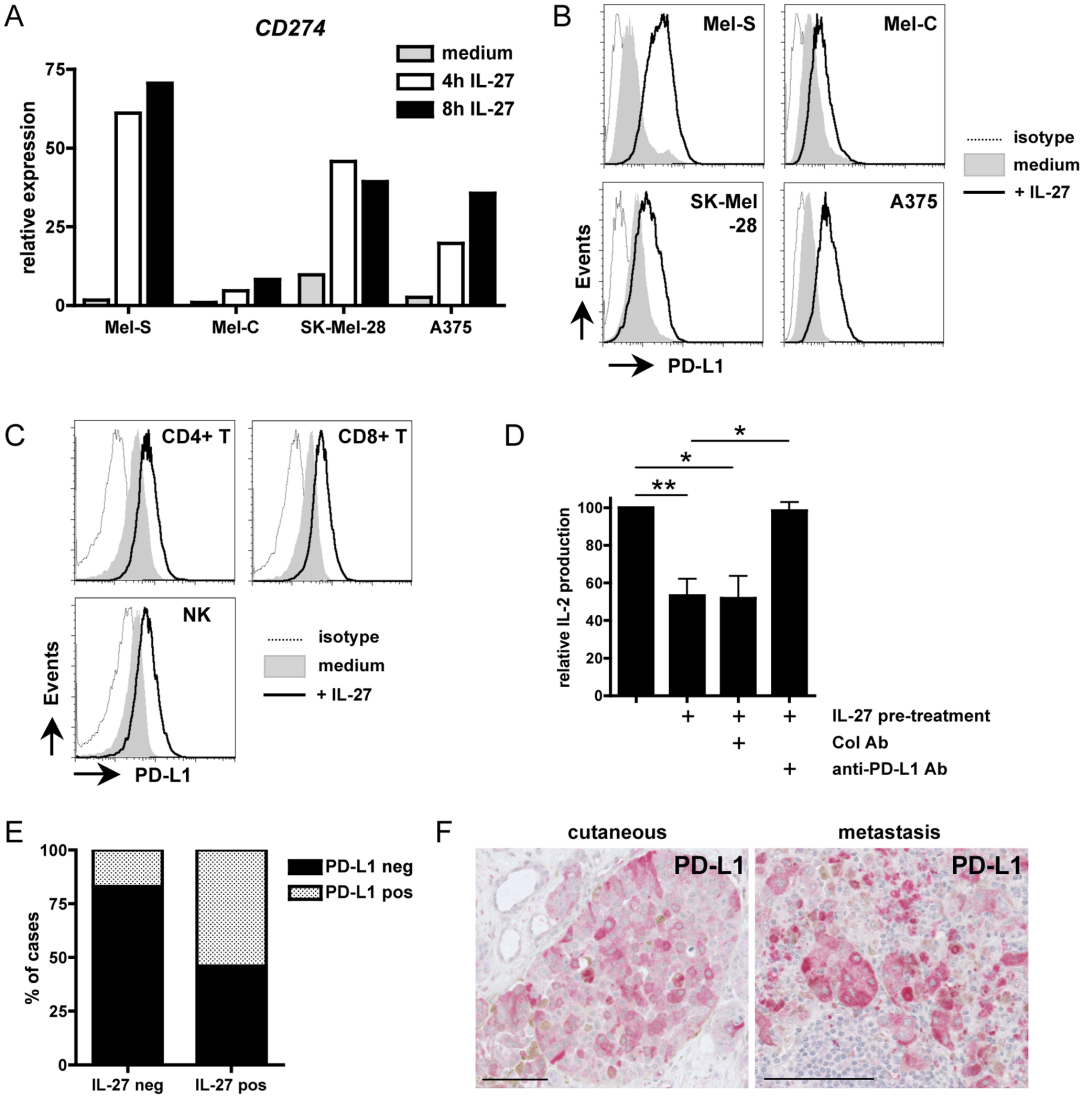
Analysis of PD-L1 induction by IL-27 in melanoma. (A) Expression of *PD-L1* (*CD274*) was analyzed by qPCR in melanoma cell lines cultured for the indicated times in the absence or presence of IL-27. (B) These cell lines were incubated for 15 hrs in the absence (medium) or presence of IL-27, stained with isotype control or anti-PD-L1 mAb and analyzed by FACS. (C) PBMC were cultured for 15 hrs in the absence or presence of IL-27. Isotype control or PD-L1 stainings were analyzed by FACS on gated CD4+ T cells, CD8+ T cells or CD16+CD56+ NK cells. A representative experiment of 2 to 3 is shown in (A-C). (D) A375 cells that had been cultured for 15 hrs in the absence or presence of IL-27 were extensively washed and incubated with PBMC and CD3/CD28 beads in the presence of Abs as indicated. After 3 days, cell culture supernatants were collected and tested by IL-2 ELISA. Results shown are mean (± SEM) of 3 independent experiments performed using 2 different donors (paired t test, * : p<0.01, ** : p<0.05). In the absence of activated PBMC, no IL-2 was detected. (E) Distribution of cases expressing or not PD-L1, among IL-27-negative or -positive cutaneous and metastatic melanomas. (F) Staining for PD-L1 in a case of a primary cutaneous melanoma and a case of metastatic melanoma that were positive for IL-27. The bar represents 100 µM.

Another suppressive molecule that was recently shown to be induced by IL-27 is the B7 family molecule, PD-L1 [32], [33]. Indeed, PD-L1 was recently shown to be induced by IL-27 on mouse CD4+ and CD8+ T cells and this induction was demonstrated to contribute to the suppressive effect of IL-27 [33]. *In situ* studies have described the expression of PD-L1 by tumor cells in melanomas [34], [35]. Therefore, we next investigated whether IL-27 could induce PD-L1 expression in melanoma cells by testing its induction in the 4 melanoma cell lines described above. In all cell lines, an induction of PD-L1 upon IL-27 treatment was detected by qPCR analysis (from 8.3 to 70.5 fold-induction after a 8 hr treatment with IL-27) (Figure 5A). This induction was confirmed by FACS analysis in melanoma cells stimulated for 15 hrs with IL-27 (Figure 5B). PD-L1 induction by IL-27 was also observed in human CD4+ and CD8+ T cells, as well as human NK cells (Figure 5C). These data indicate that IL-27, produced locally, could induce PD-L1 expression on both melanoma cells and reactive immune cells.

Binding of PD-L1 to the PD-1 receptor inhibits T-cell activation. To analyze whether IL-27-mediated PD-L1 induction on melanoma cells could have an immunosuppressive effect, we performed co-culture experiments in which A375 cells were pre-treated or not with IL-27 for 15 hrs before incubation with PBMC and CD3/CD28 beads for 3 days. Pre-treatment with IL-27 resulted in lower IL-2 production in the co-culture that was specifically reversed by addition of an anti-PD-L1 neutralizing Ab (Figure 5D), indicating that PD-L1 induction by IL-27 on melanoma cells decreased T-cell activation as expected.

We next investigated whether PD-L1 expression by melanoma cells correlated with IL-27 expression by tumor cells *in situ*. PD-L1 detection on paraffin-embedded tissues has been shown to be trickier than on frozen tissues and to underestimate the numbers of PD-L1-positive cases [35]. Of the 40 cases of invasive primary cutaneous melanoma or metastatic melanoma that were tested for PD-L1 by immunohistochemistry, only 17 (43%) showed clearcut PD-L1 staining. These PD-L1-positive cases were enriched among IL-27-positive cases compared to IL-27-negative cases. Indeed, while 15/28 (54%) of IL-27-positive cases were also positive for PD-L1 in tumor cells, only 2/12 (17%) of IL-27-negative cases stained positive for PD-L1 (p<0.05) (Figure 5E and F).

## Discussion

This study extends our knowledge on the expression profile and potential role of IL-27 in human pathologies. We and others have previously shown that IL-27 is expressed at high levels in various Th1 and/or chronic inflammatory conditions such as tuberculosis, sarcoidosis, inflammatory bowel diseases, visceral leishmaniasis, and rheumatoid arthritis. In these conditions, the main sources of IL-27 were cells of the myeloid lineage [22], [36], [37].

While many studies have investigated the role of ectopic expression of IL-27 in murine tumor models, little is known on the role of endogenous IL-27/IL-27R in the development of human tumors. In previous analyses, we observed that one subunit of IL-27, EBI3, was expressed at high levels by tumor cells in specific forms of lymphomas such as Hodgkin lymphoma, EBV or HTLV1-associated lymphoproliferative diseases and diffuse large B-cell lymphomas. However, we failed to detect p28 co-expression in most cases [23], [38]–[40]. In patients with acute myeloid leukemia, IL-27Rα has been linked to transformation through its ability to dimerize and to constitutively activate a mutant form of Jak2 [41].

In this study, we show that tumor cells in melanoma can co-express both subunits of IL-27. Several lines of evidence suggest that IL-27 expression by tumor cells may not have an adverse outcome on tumor progression, but instead may favor this progression. First, IL-27 expression profile correlates with tumor progression : whereas IL-27 is not expressed in benign melanocytic lesions nor in the least aggressive forms of malignant melanoma, it is expressed at a higher frequency in advanced stages of primary cutaneous melanomas and this expression is maintained in metastatic lesions. Moreover, IL-27-positive invasive cutaneous melanoma cases exhibit a higher frequency of metastasis compared to IL-27-negative cases.

IL-27 can act both as a positive or negative regulator of immune responses and the balance between both types of effects depends on multiple factors. We found that IL-27 expression correlates with the *in situ* expression of two immunosuppressive molecules, IL-10 and PD-L1, that are potent T-cell inhibitors. In addition, in *in vitro* experiments using human melanoma cell lines, we showed that IL-27 can induce the expression of IL-10 and, more consistently, that of PD-L1 in these cells, suggesting that an autocrine loop may operate in these cases. The murine B16F10 melanoma cell line used in mouse models does not express the IL-27Rα chain and does not respond to IL-27 [42]. Thus, the autocrine effect of IL-27 on tumor cells could not be observed in previous studies, which may in part account for the discrepancy between our *in situ* observations and the findings obtained using this murine model. Differences between human and mouse IL-27 biological effects may also account for our findings [6]. Indeed, such differences have been previously described, in part because of different regulation of IL-27 receptor expression [43]. Altogether, our data question the use of recombinant IL-27 in anti-melanoma therapy in humans.

It is interesting to note that IL-27 is expressed at very high levels in human placenta, a site of immune tolerance [5], [44]. Placental cells and tumor cells share common mechanisms to escape the immune response. These include the expression of suppressive molecules, among which IL-10 and PD-L1 [45]. Their expression of IL-27 might constitute an additional mechanism to evade the immune response.

The PD1/PD-L1 pathway has emerged as a central player in immune regulation and cancer cells that express PD-L1 promote tumor progression through inhibition of PD-1 expressing effector cells. The importance of the PD1/PD-L1 axis in the development of melanoma has been highlighted by recent clinical trials. Patients with advanced melanoma treated with anti-PD1 or anti-PD-L1 Abs showed an objective response in 28% and 17% of cases, respectively, the highest percentages among the various tumors tested [46], [47]. Patients that exhibited an objective response had all tumor cell expression of PD-L1 [47]. So far, the physiopathological role of PD-L1 induction by IL-27 has been studied only in the context of experimental autoimmune encephalomyelitis, where PD-L1 induction results in inhibition of Th17 cell differentiation and reduced pathology [33]. The role of PD-L1 induction by IL-27 in the context of tumorigenesis needs further investigation.
