# Correction: Expression of IL-27 by Tumor Cells in Invasive Cutaneous and Metastatic Melanomas

**DOI:** 10.1371/annotation/e1a0e85e-e632-40f2-9925-e0b71eb18b56

**Published:** 2013-11-07

**Authors:** Julie Gonin, Agnès Carlotti, Céline Dietrich, Anne Audebourg, Brigitte Radenen-Bussière, Anne Caignard, Marie-Françoise Avril, Marie-Cécile Vacher-Lavenu, Frédérique Larousserie, Odile Devergne

The title of the article is incorrect. The correct title is: "Expression of IL-27 by Tumor Cells in Invasive Cutaneous and Metastatic Melanomas." The correct citation is: Gonin J, Carlotti A, Dietrich C, Audebourg A, Radenen-Bussière B, et al. (2013) Expression of IL-27 by Tumor Cells in Invasive Cutaneous and Metastatic Melanomas. PLoS ONE 8(10): e75694. doi:10.1371/journal.pone.0075694. 

